# Understanding Resilience and Mental Well-Being in Southwest Indigenous Nations and the Impact of COVID-19: Protocol for a Multimethods Study

**DOI:** 10.2196/44727

**Published:** 2023-07-13

**Authors:** Julie A Baldwin, Angelica Alvarado, Karen Jarratt-Snider, Amanda Hunter, Chesleigh Keene, Angelina E Castagno, Alisse Ali-Joseph, Juliette Roddy, Manley A Begay Jr, Darold H Joseph, Carol Goldtooth, Carolyn Camplain, Melinda Smith, Kelly McCue, Andria B Begay, Nicolette I Teufel-Shone

**Affiliations:** 1 Northern Arizona University Flagstaff, AZ United States

**Keywords:** community-engaged research, Indigenous, Native nations, COVID-19, resilience, well-being

## Abstract

**Background:**

Despite experiencing many adversities, American Indian and Alaska Native populations have demonstrated tremendous resilience during the COVID-19 pandemic, drawing upon Indigenous determinants of health (IDOH) and Indigenous Nation Building.

**Objective:**

Our multidisciplinary team undertook this study to achieve two aims: (1) to determine the role of IDOH in tribal government policy and action that supports Indigenous mental health and well-being and, in turn, resilience during the COVID-19 crisis and (2) to document the impact of IDOH on Indigenous mental health, well-being, and resilience of 4 community groups, specifically first responders, educators, traditional knowledge holders and practitioners, and members of the substance use recovery community, working in or near 3 Native nations in Arizona.

**Methods:**

To guide this study, we developed a conceptual framework based on IDOH, Indigenous Nation Building, and concepts of Indigenous mental well-being and resilience. The research process was guided by the Collective benefit, Authority to control, Responsibility, Ethics (CARE) principles for Indigenous Data Governance to honor tribal and data sovereignty. Data were collected through a multimethods research design, including interviews, talking circles, asset mapping, and coding of executive orders. Special attention was placed on the assets and culturally, socially, and geographically distinct features of each Native nation and the communities within them. Our study was unique in that our research team consisted predominantly of Indigenous scholars and community researchers representing at least 8 tribal communities and nations in the United States. The members of the team, regardless of whether they identified themselves as Indigenous or non-Indigenous, have many collective years of experience working with Indigenous Peoples, which ensures that the approach is culturally respectful and appropriate.

**Results:**

The number of participants enrolled in this study was 105 adults, with 92 individuals interviewed and 13 individuals engaged in 4 talking circles. Because of time constraints, the team elected to host talking circles with only 1 nation, with participants ranging from 2 to 6 in each group. Currently, we are in the process of conducting a qualitative analysis of the transcribed narratives from interviews, talking circles, and executive orders. These processes and outcomes will be described in future studies.

**Conclusions:**

This community-engaged study lays the groundwork for future studies addressing Indigenous mental health, well-being, and resilience. Findings from this study will be shared through presentations and publications with larger Indigenous and non-Indigenous audiences, including local recovery groups, treatment centers, and individuals in recovery; K-12 and higher education educators and administrators; directors of first responder agencies; traditional medicine practitioners; and elected community leaders. The findings will also be used to produce well-being and resilience education materials, in-service training sessions, and future recommendations for stakeholder organizations.

**International Registered Report Identifier (IRRID):**

DERR1-10.2196/44727

## Introduction

### Background

The widespread COVID-19 pandemic has tested the limits of medical resources and public health, moved society toward a more isolated lifestyle, and challenged the lives of many, including several heavily impacted populations [1]. At the time this project started (2020), some of the populations most affected by COVID-19 in the United States were living in the 22 sovereign Native nations in Arizona. More than 103,000 cases of COVID-19 were diagnosed in the citizens of Native nations in Arizona, accounting for 50% of the cases in some counties [2]. Compared with other races and ethnicities in the United States before the COVID-19 pandemic, American Indian and Alaska Native populations (note that we use the terms American Indian and Alaska Native, Native American, Indigenous, and Native for this paper interchangeably to refer to Indigenous Peoples of the United States) were disproportionally affected by illnesses and chronic diseases and had an overall lower life expectancy [3]. Despite experiencing many adversities, American Indian and Alaska Native populations have demonstrated tremendous resilience during the COVID-19 pandemic, drawing upon Indigenous determinants of health (IDOH) and Indigenous Nation Building [4].

### Purpose

Our multidisciplinary team of scholars sought to implement the study described in this paper to achieve two aims: (1) to determine the role of IDOH in tribal government policies and actions that support Indigenous mental health and well-being and, in turn, resilience during the COVID-19 crisis; and (2) to document the impact of IDOH on Indigenous mental health, well-being, and resilience of 4 specific community groups—first responders, educators, traditional knowledge holders and practitioners, and the substance use recovery community living or working (or both) in or near 3 Native nations in Arizona. The overarching research question for the study was as follows: What are the IDOH in Native nations and communities that shape mental health and well-being and, in turn, resilience during the COVID-19 pandemic? We posited that the use of IDOH, an Indigenous Nation-Building framework [5,6], and complementary data collection methods would be useful for understanding the mental health needs, assets, and resources of Native nations during the COVID-19 crisis to best formulate future research, practices, and policy initiatives [6]. Native nations have maintained a sense of autonomy as sovereign entities and have responded to the pandemic by leveraging community assets informed by language, land, history, and ceremony [7]. This research was designed to guide tribal programs to improve the mental health and well-being of Indigenous Peoples in Arizona, especially during tumultuous times such as a pandemic.

### Conceptual Framework

**Figure 1:**
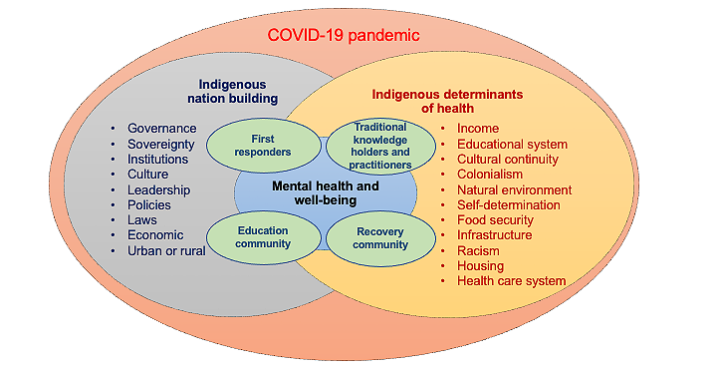
Conceptual framework.

To guide this study, we developed a conceptual framework (Figure 1) based on the IDOH, Indigenous Nation Building, and concepts of Indigenous mental well-being and resilience. Social determinants of health (SDOH) refer to conditions in the environments where people are born, live, learn, work, play, worship, and age that affect a wide range of health, functioning, and quality-of-life outcomes and risks [8]. Indigenous Peoples have unique historical, political, and social contexts that also affect health and differ from the traditional definitions of the SDOH. IDOH includes a wide variety of physical, social, and cultural conditions that can affect the health status of Indigenous Peoples [4,9]. Poverty, racism, geographic location, and education systems are examples of social impacts on the health and life expectancy of American Indian and Alaska Native populations that can put them at an increased risk for COVID-19 [9-12].

Indigenous Nation Building refers to “the political, legal, spiritual, educational, and economic processes through which Indigenous Peoples engage in order to build local capacity to address their educational, health, legal, economic, nutritional, relational, and spatial needs” [13-15]. These processes are an assertion of the federally recognized sovereignty of the Native nations. Sovereignty, applied to federally recognized Indigenous nations in the United States, means that such nations are distinct political entities with the right of self-government. Recognized through treaties, acts of congress, and presidential executive orders, Indigenous nations have a government-to-government relationship with the federal government [16,17]. During the pandemic, Native nations took the steps necessary to ensure the protection of their citizens from the threats of COVID-19 with the implementation of stay-at-home orders; weekend lockdowns (restricting citizens from leaving their communities); and the closure of businesses, establishing vaccination centers, mask mandates, and other preventive recommendations. The assertion of sovereignty serves as an important means of managing the risks to citizens’ health, a mechanism for maintaining adequate sociocultural strength, health, and well-being in Indigenous populations [18].

The concept of Indigenous mental health and well-being is receiving increased attention in the literature as scholars uncover the unique stressors that Indigenous Peoples face as well as the culturally distinct approaches that Indigenous nations and communities use to support resilience [19-21]. A key component of Indigenous mental health and well-being is the recognition of the impact of historical trauma. Historical trauma is a cumulative, multigenerational experience of trauma that impacts emotional and psychological well-being from one generation to the next and can disrupt the strength of the community in Native nations [22,23]. The impact of historical trauma is reflected in American Indian and Alaska Native populations using drugs and alcohol at younger ages and at higher rates than other populations [24-27], reporting serious psychological distress at higher rates [28], and committing suicide at younger ages than non–American Indian and Alaska Native populations [29]. Historical trauma affects Indigenous populations and manifests in many ways, including by impacting Indigenous mental health and well-being outcomes. Even with historical trauma, American Indian and Alaska Native populations have maintained their resilience through the continuation of ceremonial practices, speaking heritage languages, passing on oral histories, and implementing local strategies to reinforce social support systems and cultural capital [30].

In the United States, approximately 4.2 million Americans identify as American Indian and Alaska Native [31]. The living conditions for American Indian and Alaska Native populations vary greatly, with 78% living off reservations and 22% living on reservation lands [32]. Regardless of residence, the cultures of American Indian and Alaska Native populations generally emphasize connections to others, the past, the natural world, and traditional homelands [21,33]. This worldview is demonstrated by strong family bonds, respect for the wisdom of elders, and the maintenance of cultural traditions. These practices preserve, maintain, protect, and serve as solutions against threats to mental health and well-being in Indigenous communities [21,34].

## Methods

### Overview

This study merged the IDOH and Indigenous Nation-Building frameworks to establish an understanding of the interrelationship of sovereignty, land jurisdiction, cultural identity, continuing effects of colonialism, and resilience that impact mental health and well-being. The research design focused on 4 groups in 3 Native nations and an urban Indigenous health center near one of these nations that have served their communities while remaining exposed to the effects of the COVID-19 pandemic or were heavily impacted by physical distancing restrictions. These groups included first responders, educators, traditional knowledge holders and practitioners, and members of the substance use recovery community.

In the analysis, mental health and well-being were not addressed from a psychological or individual clinical orientation but through a contextual and external environmental lens, otherwise understood as a systems orientation [35]. A systems orientation has been used in understanding perspectives within human service program systems and the relationship of organisms in their natural habitat, but to our knowledge, it has never been used to investigate mental health and well-being in Native communities [36].

### Indigenous Guided Research

The research process was guided by the Collective benefit, Authority to control, Responsibility, Ethics (CARE) principles for Indigenous Data Governance to honor tribal and data sovereignty [37-39]. The CARE principles ensure that, when working with external research partners, Native nations receive the following: (1) collective benefit of research findings, (2) authority to control data, (3) responsible research partners who support self-determination, and (4) ethical research partners who honor the rights and well-being of sovereign nations and their community members. Individuals from 3 Native nations and an urban Indigenous health center in Arizona participated in this study with approval from their institutional review board (IRB), Tribal Council, or Cultural Preservation Office. The research approval process spanned 7 months (June 2020 to January 2021) and was preceded by multiple years of relationship building through research and service between members of the research team and each of the 3 nations. Our study was unique in that our research team consisted predominantly of Indigenous scholars representing at least 8 tribal communities and nations in the United States. The members of the team, regardless of whether they identified themselves as Indigenous or non-Indigenous, have many collective years of experience working with Indigenous Peoples and are committed to the health and well-being of tribal communities. A community researcher from each Native nation was hired as a vital member of our research team to aid in recruiting participants, scheduling interviews and talking circles, translating interviews from the Native language into English, and providing overall expertise to ensure that methodologies and approaches were culturally congruent and respectful.

### Research Design

**Figure 2:**
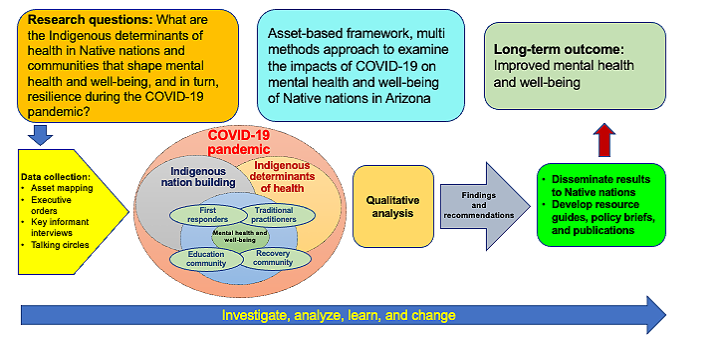
Research methodology.

The team investigated the resilience and contextual factors that either facilitated or hindered these efforts. The goal of understanding resilience was informed by the Indigenous Nation-Building framework [14,40]. As depicted in Figure 2, data were collected through a multimethods research design that documented the contextual factors that contribute to mental health and well-being among the people of the Native nations. Contextual factors included educational systems, socioeconomic status, access to food and health care, and systemic oppression. Special attention was placed on the assets and culturally, socially, and geographically distinct features of each Native nation and the communities within them. Indigenous Peoples and nations demonstrated resilience in many ways during this difficult period. American Indian and Alaska Native populations have experienced substantial and unique challenges during the pandemic, including increased levels of anxiety, stress, and depression. The team explored these emotions and resilience strategies through interviews and talking circles. Figure 2 provides an overview of the research questions, data collection methods, and the expected outcomes. This paper presents our methodological protocol for each aim.

### Aim 1: Determine the Role of IDOH in Tribal Government Policy and Action That Support Indigenous Mental Health, Well-Being, and, In Turn, Resilience During the COVID-19 Crisis

#### Asset Mapping

Community asset–based mapping is a process that actively seeks the best use of community resources and creates a profile of existing buildings, programs, and services in the community [41,42]. Our team began the study by searching Google Maps, publicly available reports, and other relevant community resources. A spreadsheet of electronically available assets was compiled, including the title or name of the asset, contact information, and type or category of the asset. Categories of assets included mental health; general health; education; cultural resources including traditional knowledge holders, economic, recreation, and government and social services; and other resources including religious and spiritual resources. The team consulted with community researchers and a select group of community members from each Native nation to confirm the accuracy and completeness of the lists. Next, the team worked with colleagues at our university’s Geographic Information System laboratory to create resource maps. The maps were created for each of the 3 Native nations, including separate maps according to the asset type or category for each nation. The research team then reviewed the full list and maps and coded each asset using a codebook developed from key terms in the conceptual framework. At least 2 researchers independently coded each nation’s asset list; any discrepancies were discussed, and a final coding was determined by the team. Finally, the team created a community resource guide for each of the 3 nations; these guides were organized by category and included both the lists and the maps of the assets identified through this phase of the study.

#### Executive Orders Coding

Executive orders, executive directives, and public health emergency orders were collected for each of the 3 participating nations from March 1, 2020, to June 15, 2022. Each executive order or directive (1 nation uses the term directives rather than executive orders) was scanned for content, and a team of 6 researchers, working in pairs, developed a codebook based on the content of all the orders. The orders were coded according to the issuing authority (ie, top-ranking executive leaders or public health authority), the topics, and any requirements of the order. For example, an executive order issued by the Native nation’s president or chairperson requiring employee vaccinations was coded by the nation, the issuing office or authority, and the code “vaccination requirement.” Each order or directive was coded by an independent researcher and verified by a second researcher. Any discrepancies as well as the need to establish new parent or child codes were discussed by the entire coding team; final decisions were reached through consensus of the team.

Federal executive orders or directives, such as a federal mask mandate, and guidance from the Centers for Disease Control and Prevention (CDC) may have informed the executive orders, directives, or public health emergency orders of the participating Native nations. For example, the CDC issued the guidance for safety measures such as social distancing, hand washing, and mask wearing, which, in turn, was followed by governments across the country. Native nations also instituted CDC-recommended safety measures and encouraged their citizens to follow them, as evidenced in the executive orders, executive directives, and public health emergency orders examined in this study. However, as federally recognized Native nations are sovereignty entities and have a legal status higher than the states [43], the state executive orders of states adjacent to or surrounding the lands and jurisdictions of the participating Native nations are not included in the coding or analysis.

Upon the completion of the coding, the analysis will begin. Using content analysis, researchers will examine the orders to determine what actions governments took or policies were developed to support the resilience and mental well-being of their citizens.

### Aim 2: Document the Impact of IDOH on Indigenous Mental Health, Well-Being, and Resilience of 4 Specific Community Groups

#### Recruitment

Recruitment parameters for participants were that they must be aged 18 years; identify as someone who works in or is a citizen of 1 of the 3 participating Native nations; and identify as a member of one of the four informant groups: (1) first responder—identify as a first responder (ie, they are associated with law enforcement, fire department, or emergency management training or work as emergency room doctors or nurses); (2) educator—identify as an educator or teacher; (3) traditional knowledge holder or practitioner—identify as a traditional medicine person or as being a part of a medical people association and have the knowledge and experience in maintaining, participating in, and practicing traditional Native ways of life; and (4) substance use recovery member—identify as a substance use recovery provider or an individual in substance use recovery who uses recovery services. Exclusion criteria were being incarcerated, incapacitated, or unwilling to provide informed consent to participate in the study activities or currently participating in a substance use recovery program (recovery group only). We prioritized recruiting individuals who identified as American Indian and Alaska Native. However, for some subgroups, providers and personnel included non-Indigenous individuals who worked within or for the participating communities. These individuals represented 13% (12/92) of all the interviewees. For the recovery community, the team also recruited from an urban Indigenous health center, Native Americans for Community Action, based in Flagstaff, Arizona.

Participants were recruited through email, in-person presentations to related entities and individuals within specified subgroups and communities, and face-to-face interactions. The research team used snowball sampling and social networks guided by community researchers’ and investigators’ community contacts.

Informed consent was obtained using multiple methods in an effort to provide flexibility as the COVID-19 situation evolved. These methods primarily included a signed informed consent form mailed to the participants, the review of an oral or web-based script via phone, and verbal consent given by the participants. If participants provided oral consent, the researchers documented the participant’s name, date of consent, and specified the conditions of consent. In some cases, oral consent was provided in the participants’ respective Indigenous language.

All subgroups (first responders, educators, traditional knowledge holders and practitioners, and members of the substance use recovery community) from each partnering community participated in key informant interviews. Only 1 Native nation and 3 subgroups (educators, members of substance recovery community, and traditional knowledge holders and practitioners) participated in talking circles. In an effort to follow the CDC guidelines for safety during the pandemic, interviews and talking circles were conducted via a password-secured Zoom link or by telephone. Talking circles were conducted after the interviews were completed.

#### Key Informant Interviews

Each participant completed an individual 1- to 2-hour interview intended to identify SDOH and the conditions that may have contributed to mental health stressors or sources of strength during the COVID-19 pandemic and IDOH contributing to stress or strength. The interviewees were given a US $25 gift card for their time. Guided by the need to obtain a representative sample, the team attempted to conduct at least 4 interviews with a goal of 10 with each subgroup from each participating nation. The protocol for the key informant interviews for all 4 subgroups was similar but had some nuances by subgroup, as described in subsequent sections for each community group. The interview questions were developed and edited by the whole team for a period of 2 to 3 months. The research team aimed to create “core” questions that could be used to analyze themes across groups and group-specific questions that allowed for the identification of group-specific challenges and solutions. Each group was asked 5 core questions, with additional questions relevant to the specific group. The 5 core questions were as follows:

1. Please talk about your tribal government’s response during the COVID-19 pandemic?
2. What does the term “mental well-being” mean to you?
3. Has your (insert Indigenous community) cultural identity affected your mental well-being during the COVID-19 pandemic? If so, how?
4. What does the term “resilience” mean to you?
5. Has your (insert Indigenous community) cultural identity affected your resilience during the COVID-19 pandemic? If so, how?

The first responder research group was interested in learning how the pandemic may have changed the workload for first responders and how changes owing to the pandemic affected them on a personal level (eg, physical, emotional, spiritual, cultural, mental, and financial levels and on the basis of family or home and food security). We were also interested in learning about the resources that were available to them and what resources they needed during the pandemic to stay healthy. Examples of group-specific questions for the first responders group were as follows:

1. Have there been any effects of COVID-19 on you personally, and if so, how? (prompts: physically, emotionally, spiritually, culturally, mentally, family, finances, and food security)
2. Has COVID-19 affected your job? If yes, how?
3. Is there anything else that could be done by people at your work to help you? (prompts: check on you, talk with you, use humor, do things different, and if so, like what?)
4. What, if any, resources are available to you to help you stay healthy? (prompts: agencies, people, and places)

The education research group was interested in understanding how educators responded to the transition from face-to-face learning to web-based learning and sometimes having to switch between the 2 modalities after in-person COVID-19 exposure. Because of the circumstances experienced by the teachers, it was particularly important for the educator research group to learn about and understand their experiences of resilience and mental well-being. Specifically, it was important to give educators time to speak about the impact of COVID-19 on them individually and on their respective professional roles in education, as well as what resources and support they accessed during that time. Another area of focus was to learn more about how the range of decision-making entities (ie, school boards, tribal governments, and federal governments) impacted their role as educators. Finally, we asked questions related to personal and classroom resources that were helpful or would have been helpful during the pandemic. The questions for this group included the following:

1. What has been the impact of COVID-19 on you? (prompts: physically, emotionally, spiritually/culturally, mentally, family, finances, and food security)
2. How has COVID-19 impacted doing your work as an educator?
  What has helped you as an educator adapt to changes caused by COVID-19?
  How have you responded to the ways in which COVID-19 has affected your families and students?
  How has your broader community/school responded to COVID-19?
3. What resources have you used during this pandemic to help you stay healthy?
  What resources have you used to support students and families?
  What resources do you wish were available to support your health and the health of students and families?
4. Tell me about the various levels of leadership that you believe impact your role as an educator.
  How have these levels responded?
  How has school/district leadership responded and how has it impacted you in your professional role?

In times of uncertainty, many Indigenous Peoples went to traditional knowledge holders and practitioners for assistance in resolving health issues, and the pandemic was no exception. Given that there were no Western medicines available to combat COVID-19 at the time, traditional knowledge holders and practitioners became especially important. The questions focused on how the patients of traditional knowledge holders and practitioners were affected by the pandemic and how they were coping. Furthermore, understanding how the pandemic might have impacted the tasks and duties of traditional knowledge holders and practitioners was critical to highlighting the role of cultural capital in resilience. We asked the following questions about how traditional knowledge holders and practitioners viewed the meaning of resilience and mental well-being:

1. Have the people you serve maintained resilience during the pandemic?
  If so, how? If not, why not?
2. Has your cultural practice been impacted by COVID-19? (prompts: physically, emotionally, spiritually/culturally, mentally, financially, and adaptations)
  If so, how? If not, why not?
3. What do you think about the changes you made?

The members of the substance use recovery research group were particularly interested in how restrictions imposed on social gatherings affected the practice of recovery for American Indian and Alaska Native populations. Although programs exist that are specifically constructed for Native American recovery (ie, The Red Road to Wellbriety and Healing of the Canoe) [44,45] and place Native American culture as central to treatment, most recovery programs that are altered to serve American Indian and Alaska Native populations use traditional practice as an added element to mainstream programs such as Alcoholics Anonymous or Narcotics Anonymous [46]. The group process, intended to remove stigma and promote social support, is an important evidence-based practice in almost every recovery program. Most American Indian and Alaska Native cultures emphasize connectedness to tribes, clans, and families. Therefore, the recovery group asked nonleading questions such as the following to understand the impact of social restrictions on recovery:

1. Please describe to me how your work has changed because of the COVID-19 pandemic.
2. Please describe to me any special qualities or circumstances that have allowed Native Americans in recovery to adapt positively to the new circumstances presented by the presence of the virus.
3. Please describe to me any special qualities or circumstances that have affected Native Americans in recovery to negatively respond to the new circumstances presented by the presence of the virus.

#### Talking Circles

The talking circle is a common and respectful way of sharing, listening, and learning among North American Indigenous Peoples [47,48]. Talking circles are also referred to as “sharing circles” by some Indigenous Peoples [47,49]. Facilitators of talking circles used open-ended questions designed to assess specific direct and indirect impacts of the pandemic and ways of adapting and responding to those impacts (resilience). Talking circles for this study lasted for 1 to 3 hours and included 2 to 6 individuals. The research team conducted 1 talking circle session with educators, 2 with substance use recovery community members, and 1 with traditional knowledge holders and practitioners, each of which consisted of members from a single participating nation. Talking circle participants were recruited through community partner agencies and by the community researchers. Each participant was given a gift card worth US $50 for their time. Each talking circle included variations in the key informant interview questions. The talking circle questions are presented in Textbox 1.

**Talking circle questions.**

**Educators**

- To the extent you feel comfortable, share the impact of COVID-19 on you.
- How has COVID-19 impacted your work as an educator (principal, counselor, teacher, etc)?
- What resources have you used during this pandemic to help you stay “healthy”?
- Tell me about the various levels of leadership that you believe impact your role as an educator.

**Substance use recovery community**

- To the extent that you feel comfortable sharing, how have you been impacted by COVID-19?
- How has your recovery been impacted by COVID-19?
- How did you adapt to and respond to the impact on your recovery? Can you identify the methods, strategies, or specific programs that supported your recovery?
- What do the terms “mental well-being” and “resilience” mean to you?

**Traditional knowledge holders**

- To the extent that you feel comfortable sharing how has your cultural practice been impacted by COVID-19?
- How have you adapted and responded to those impacts?
- What methods or strategies have you used during this pandemic in your role as a practitioner/knowledge holder to support people?

#### Coding and Analysis

With participant permission, interviews and talking circles were audio recorded and transcribed verbatim for analysis. One subgroup transcribed the audio recordings from Native language to English for analysis. For Zoom sessions, participants had a choice to turn their video on or off during the interviews and talking circles. If a participant did not want their name to be displayed on the video screen, the community researcher helped the participant change their name to an alias name on the screen. If the participants chose to keep their video on, they were reminded that all video and audio sessions would be recorded. Once the audio segments from all sessions were saved, the video portions were deleted. If a participant did not wish to be audio recorded, then a researcher (not the facilitator) took thorough notes for further analysis. The research team deidentified all data, including any tribal affiliations or agency names before further analysis because participating Native nations requested that data should not be identified by the tribal communities. Participants were assigned a unique ID code to remove any connections to their personal identifying information. No identifying information was included in the descriptive or analytical result tables or reports.

Once interviews and talking circles were completed, the research team conducted quality checks of the audio recordings to ensure that the transcriptions were accurate and to eliminate any identifying information before analysis. A secondary codebook (similar to the asset mapping codebook) was created based on the conceptual framework presented in the *Introduction* section. Members of the research team, including researchers from American Indian and Alaska Native nations, regularly met to discuss the definitions and applications of the codes. After developing the initial draft of the codebook, all members of the coding team coded the same transcript to compare coding and address any issues with the codebook. The codebook was refined when new themes emerged. The codebook consisted of 30 codes and child nodes that were entered into the NVivo software (NVivo 12 Pro; Lumivero). One team member was appointed to create and manage the NVivo master project that was used to house the master codebook. The NVivo software was used to categorize quotes with codes to perform qualitative analyses of the topics discussed in both the interviews and talking circles.

Because of the large number of transcripts, each transcript was coded by 1 coder, and interrater reliability was not calculated. Regular coder debriefing sessions, a highly structured codebook, and the purposeful discourse and integration of Indigenous and non-Indigenous interpretations of narratives ensured the reliability of the coding process [50,51]. All the coders were involved in codebook development, which included biweekly meetings for the duration of the coding process. Biweekly coding meetings allowed coders to ask for clarification on the application of the codes in the context of the interviews they were coding. This ongoing communication supported and built a consensus on the application of the codes and child nodes. In addition, as alternate interpretations of a code were discussed, analysis was guided in the codebook by the addition of inclusionary and exclusionary examples extracted from the narratives to yield agreement in the coding process [50].

This triangulation of methods (interviews, talking circles, asset mapping, and coding of executive orders) and data (eg, narratives, maps, and categories of orders) informed our understanding of the context and strategies of American Indian and Alaska Native populations and Native nations and overall research credibility. Triangulation was further strengthened through coders working in teams and through the biweekly meetings of the entire coding team.

### Ethics Approval

The study was reviewed and approved by IRBs and entities that provide research oversight for the university and each of the participating Native nations (Northern Arizona University approval 1693297). Informed consent was obtained from the participants for both the interviews and talking circles that were conducted. This process has been described in the Indigenous Guided Research section. The study data were anonymized or deidentified. Individuals participating in the interviews received US $25 gift cards and those participating in the talking circles received US $50 gift cards.

## Results

The number of participants enrolled in this study was 105 adults, with 92 individuals interviewed and 13 individuals engaged in 4 talking circles (Table 1). Although the goal was to interview 10 individuals from each subgroup (ie, first responders, educators, traditional knowledge holders and practitioners, and members of substance use recovery community) from each of the 3 nations, this number was not consistently achieved.

The number of interview participants in each subgroup and each community ranged from 4 to 10. Because of time constraints and challenges bringing people together during the pandemic, both via web-based platforms and in person, the team elected to host talking circles with only 1 nation, with participants ranging from 2 to 6 people. Because of the limited availability of participants, a talking circle was not conducted with the first responder subgroup.

The participant demographics are presented in Table 2. In total, 63% (58/92) of the participants identified as female and 87% (80/92) identified as Indigenous. Participant age was evenly distributed among 3 age ranges: 35 to 44 years (25/92, 27%), 45 to 54 years (25/92, 27%), and 55 to 64 years (20/92, 22%). In total, 9% (8/92) of participants were in the age range of 25 to 34 years, and 15% (14/92) of participants were aged 65 years or older.

Currently, we are in the process of conducting a qualitative analysis of the transcribed narratives from interviews, talking circles, and executive orders. These processes and outcomes will be described in future studies.

**Table 1:** Number of participants in interviews and talking circles.

Community		Nation A	Nation B	Nation C	Urban^a^	Total
**Key informant interviews (number of participants interviewed/goal), n/N**						
	First responders	10/10	8/10	7/10	—^b^	25/30
	Educators	10/10	9/10	7/10	—	26/30
	Traditional knowledge holders and practitioners	10/10	6/10	6/10	—	22/30
	Members of substance use recovery	1/10	4/10	8/10	6/10	19/40
	Subtotals by community	31/40	27/40	28/40	6/10	92/130
**Talking circles (participants), n (n)**						
	First responders	0 (0)	—	—	—	0 (0)
	Educators	1 (2)	—	—	—	1 (2)
	Traditional knowledge holders and practitioners	1 (6)	—	—	—	1 (6)
	Members of substance use recovery	2 (5)	—	—	—	2 (5)
	Subtotals by community	4 (13)	__	__	__	4 (13)

^a^Participants recruited from an urban Indigenous health center in Arizona.

^b^Data not available.

**Table 2:** Demographics of key informants by nation (N=92).

Community		Nation A (n=31), n	Nation B (n=27), n	Nation C (n=28), n	Urban^a^ (n=6), n	Total, n (%)
**Gender**						
	Female	16	19	18	5	58 (63)
	Male	15	7	10	1	33 (36)
	No response	0	1	0	0	1 (1)
**Ethnicity**						
	Indigenous	30	26	19	5	80 (87)
	Non-Indigenous	1	1	9	1	12 (13)
**Age range (years)**						
	25-34	4	1	2	1	8 (9)
	35-44	5	10	9	1	25 (27)
	45-54	7	6	9	3	25 (27)
	55-64	4	7	8	1	20 (22)
	65 or older	11	3	0	0	14 (15)

^a^Participants recruited from an urban Indigenous health center in Arizona.

## Discussion

### Principal Findings

This study embraced community-engaged research approaches to determine the role of IDOH and Indigenous Nation Building in supporting American Indian and Alaska Native mental health and well-being and, in turn, resilience during the COVID-19 crisis. The study documented the impact of COVID-19 and the resilience of 4 specific community groups of 3 Native nations and an urban Indigenous health center near one of these nations, including first responders, educators, traditional knowledge holders and practitioners, and members of the substance use recovery community. The IDOH and Indigenous Nation-Building frameworks allowed the study team to acknowledge the COVID-19 protocols implemented by sovereign nations and governing leaders to use resources and take actions needed to mitigate the impact of the pandemic. The collection and analysis of tribal policy documents, communications, actions, and available assets allowed our team to understand the communities’ networks, infrastructures, and perspectives.

Engagement with members of each nation’s subgroups was key to understanding the Indigenous concepts of well-being linked to collective mental health and resilience. The communication between researchers and citizens of the collaborating nations was culturally adapted to create a trusted relationship between the research team and the sovereign nations [52]. Indigenous communities have been harmed by unethical research practices in the past [52]. Developing culturally respectful research protocols renews confidence in future ethical research and aids in the protection of the sovereign nations [52]. Other practices included ensuring confidentiality of information, receiving explicit consent for participation and recording interviews, encouraging individuals to communicate their answers in their Native language, and providing mental health resource lists for each participating community. The team conducted talking circles rather than focus groups, as they were deemed more culturally congruent than focus groups in this population. For many Native communities, talking circles provide a sacred environment for mutual respect in the sharing of personal observations, often pertaining to health disparities and actions that affect them within their tribal community [47-49]. In this study, talking circles were used to observe the communication of community members in discussing the impact of COVID-19 on their mental well-being and subsequent resilience.

By working alongside our university IRB, tribal IRBs, tribal councils, Cultural Preservation Offices, and tribal leaders, the team was able to ensure cultural sensitivity throughout the multimethods design and analyses of data. As sovereign nations, all research taking place within a Native nation must be approved by a tribal research review board, a tribal council, or a cultural review board. The research guidelines include ensuring benefits to the nation and its citizens and the nation’s ownership of the data gathered. Regular communication and engagement with each nation—including, but not solely, the community researcher of each nation—ensured respectful interaction with tribal members and established their values and rights as citizens of sovereign nations in our research.

Our study was unique in that our research team consisted of predominantly Indigenous scholars representing at least 8 tribal communities and nations in the United States and other non-Indigenous colleagues, collectively with many years of experience working with Indigenous Peoples. The development of this team was not happenstance but intentionally crafted because the team members understood the importance of bringing together people with the right expertise, identity, and relationship. The team included 1 community researcher from each of the 3 nations; these individuals lived and worked within the communities and had multiple relationships with the community members. Although other members of the research team also had long-established relationships with the Native nations, the inclusion of individuals who lived, worked, and interacted with the immediate local community was a critical component of the research design. These individuals not only played important roles in the recruitment of study participants but also provided key insights into the data analysis and interpretation of findings. Overall, our team’s background and experience were vital to the development of socially conscious decisions in the methods used throughout the study.

Findings from this study will be shared through presentations, papers, and publications with larger Indigenous and non-Indigenous audiences, including collaborating and stakeholder organizations; local recovery groups, treatment centers, and individuals in recovery; K-12 and higher education educators and administrators; directors of first responder agencies; traditional medicine people; and elected community leaders. Data and results will be used to produce resource maps to promote service availability; advocate for research and resource expansion in the region; and be disseminated at local, regional, and national conferences and advocacy events as well as peer-reviewed journals. The findings will be used to produce well-being and resilience education materials, such as print and digital toolkits, in-service training sessions, educational materials specific to Indigenous audiences, and future recommendations for stakeholder organizations. The pending articles will consist of data analysis and results pertaining to asset mapping analysis, executive orders, and the development of a codebook. In addition, each of the 4 subgroup interviews and talking circles will be analyzed for existing patterns to address present community needs and potentially new needs that have emerged from the COVID-19 pandemic.

### Limitations of the Study

This study included a small subset of 3 nations from 570 federally recognized tribal nations. Furthermore, the study was limited to Native nations within 1 geographic region of the US Southwest. In addition, among the 4 identified subgroups of the participating tribal nations, the number of interview respondents and talking circle participants was low. Finally, not all study participants were American Indian and Alaska Native.

These limitations may have implications for the results. For example, very remotely located tribal communities in other areas of the country or tribal nations with differently organized leadership may have experienced COVID-19 dissimilarly than the individuals from Native nations participating in this study; therefore, a similar study conducted in a different area of the country might yield very different results than this study.

This study examined questions through a framework of Indigenous Nation Building and IDOH, which the research team determined to be the most appropriate framework for examining the issue. Other studies using a less Indigenous-centered approach could yield somewhat different findings.

### Conclusions

In conclusion, this community-engaged study lays the groundwork for future studies addressing Indigenous mental health, well-being, and resilience in other communities during global crises. Asset mapping resource guides can create more opportunities for resource expansion in each participating community. Geographic Information System mapping can be further used for spatial analysis and to understand the distribution and availability of key resources in Native lands. Future intervention-based studies can also address mental well-being to enhance community resilience. The intentionality of linking Indigenous researchers with Indigenous communities to address pressing matters related to IDOH exemplifies the value and importance of the practice to build trust and mutual respect among communities, Native nations, and the academic community; it also reflects the 4 R’s (respect, relationship, reciprocity, and responsibility) approach described by Brayboy et al [14], which is crucial to Indigenous research methodologies. Furthermore, Indigenous Nation Building is centered in our multifaceted and interdisciplinary approach to learn from each stakeholder group toward informing tribal sovereignty and responding to a pandemic crisis through the lens of the Indigenous community. This commitment was clearly demonstrated in 1 meeting with Indigenous elders, where the elders explicitly communicated in the Native language their appreciation for this research that included a researcher who knows the language and knows the people.

## Abbreviations

CARE: Collective benefits, Authority to control, Responsibility, Ethics
CDC: Centers for Disease Control and Prevention
IDOH: Indigenous determinants of health
IRB: institutional review board
SDOH: social determinants of health

## Data Availability

The data sets generated and analyzed during this study are not publicly available because they include data from participants of sovereign Native nations, and these data cannot be released without explicit permission from each nation. Please feel free to contact the principal investigator, Dr Julie Baldwin, if you have questions.
